# Varus alignment of the proximal tibia is associated with structural progression in early to moderate varus osteoarthritis of the knee

**DOI:** 10.1007/s00167-019-05840-5

**Published:** 2020-01-21

**Authors:** Jonathan S. Palmer, Luke D. Jones, A. Paul Monk, Michael Nevitt, John Lynch, David J. Beard, M. K. Javaid, Andrew J. Price

**Affiliations:** 1 Orthopaedic Department, Dorset County Hospital, Dorchester, DT1 2JY UK; 2 Orthopaedic Department, Chelsea and Westminster Hospital, London, SW10 9NH UK; 3 Auckland Bioengineering Institute, University of Auckland, Auckland, 1142 New Zealand; 4 San Francisco Coordinating Center, University of California, San Francisco, USA; 5 Nuffield Department of Orthopaedics Rheumatology and Musculoskeletal Sciences, University of Oxford, Old Rd, Oxford, OX3 7LD UK

**Keywords:** Osteoarthritis, Knee, Mechanical alignment, Coronal, Proximal tibial angle, MPTA

## Abstract

**Purpose:**

Lower limb malalignment is a strong predictor of progression in knee osteoarthritis. The purpose of this study is to identify the individual alignment variables that predict progression in early to moderate osteoarthritis of the knee.

**Method:**

A longitudinal cohort study using data from the Osteoarthritis Initiative. In total, 955 individuals (1329 knees) with early to moderate osteoarthritis (Kellgren-Lawrence grade 1, 2 or 3) were identified. All subjects had full-limb radiographs analysed using the Osteotomy module within Medicad^®^ Classic (Hectec GMBH) to give a series of individual alignment variables relevant to the coronal alignment of the lower limb. Logistic regression models, with generalised estimating equations were used to identify which of these individual alignment variables predict symptom worsening (WOMAC score > 9 points) and or structural progression (joint space narrowing progression in the medial compartment > 0.7mm) over 24 months.

**Results:**

Individual alignment variable were associated with both valgus and varus alignment (mechanical Lateral Distal Femoral Angle, Medial Proximal Tibial Angle and mechanical Lateral Distal Tibial Angle). Only the Medial Proximal Tibial Angle was significantly associated with structural progression and none of the variables was associated with symptom progression. The odds of joint space narrowing progression in the medial compartment occurring at 24 months increased by 21% for every one degree decrease (more varus) in Medial Proximal Tibial Angle (*p* < 0.001)

**Conclusions:**

Our results suggest that the risk of structural progression in the medial compartment is associated with greater varus alignment of the proximal tibia.

**Level of evidence:**

Level III, retrospective cohort study.

## Introduction

Early to moderate knee osteoarthritis (OA) is common, hard to treat and can be debilitating for symptomatic individuals [13]. These patients are said to be in a “treatment gap” [14] where effective therapeutic interventions are limited.

A clear understanding of the predictors that cause structural progression, symptom worsening or non-progression more likely in individuals with symptomatic early to moderate knee OA is necessary.

Longitudinal cohort studies have confirmed that lower limb malalignment is a potent predictor of both incidence and progression of knee OA [9, 18, 20]. The term 'constitutional varus' has been used to describe the varus deformity seen in healthy individuals [2]. More recently, a wide variation in femoral and tibial alignment has been demonstrated in the healthy adult population [11]. Consequently, it has been suggested that traditional coronal alignment descriptions of neutral, varus and valgus using the hip–knee–ankle angle may oversimplify what is a highly variable characteristic with multiple possible phenotypes [10].

With this in mind, a longitudinal cohort study was designed using data from the Osteoarthritis Initiative (OAI). The aim was to identify whether the individual alignment variables that contribute to coronal alignment of the lower limb have a uniform effect on symptom and/or structural progression in symptomatic early to moderate knee OA. In other words, the study aimed to determine whether traditional alignment descriptions of varus, valgus and neutral are sufficient to identify patients with knee OA who are at risk of progression or is a more detailed description of coronal alignment required.

Our hypothesis was that traditional alignment descriptions oversimplify coronal alignment and that the individual alignment variables contributing to coronal alignment will have varying effects on progression of knee OA.

## Material and methods

### Study sample

The study population comprised participants from the OAI. The OAI is a multi-centre, longitudinal, observational cohort study focusing primarily on the natural history of knee OA [15].

Eligible subjects had at least one knee with Kellgren–Lawrence (KL) grade 1, 2 or 3 and symptoms in the same knee at recruitment. Symptoms were defined as knee pain, aching or stiffness: more than half the days of a month in the past year in the same knee. If a subject had two eligible knees, then both knees were enrolled in the study. To be eligible for the study, both clinical and radiographic outcomes at 24 months had to be available. All individuals had to have a full-limb radiograph (FLR) with all landmarks clearly visible and available for analysis.

**Fig. 1:**
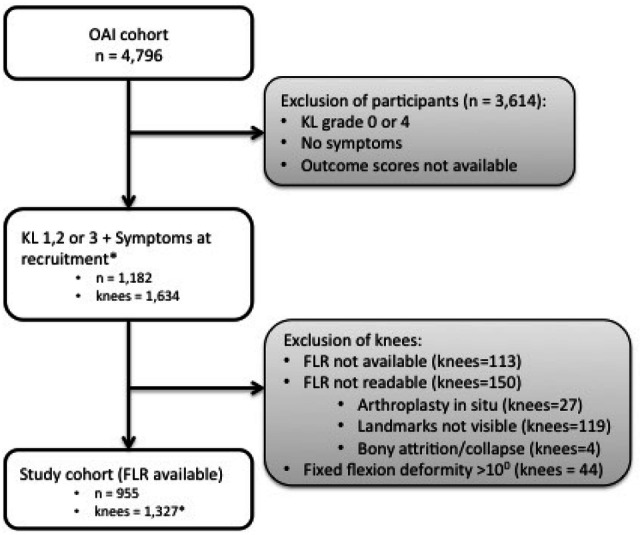
Formation of the study cohort. Flowchart illustrates the formation of the study cohort. *Timepoint at which FLRs were taken; 12 months (*n* = 965), 24 months (*n* = 230), 36 months (*n* = 112), 48 months (*n* = 20)

The OAI did not perform FLRs on any individuals at recruitment. Individuals with established OA (KL grade 2 or more and symptoms) had an FLR at 12 months. FLRs were acquired after 12 months in the remaining cohort including those subjects with KL grade 1 at recruitment (see Fig. 1).

Subjects with a significant fixed flexion deformity (FFD > 10°) at recruitment were excluded, as a small degree of rotation in such individuals can significantly affect the accuracy of coronal alignment measurements [19]. As no FLRs were taken at recruitment, some subjects demonstrated progression of OA on their FLR such that bony attrition was present on either the tibial plateau or femoral condyle. These subjects were excluded as such intra-articular deformity may have exaggerated any malalignment. Similarly, subjects were also excluded if a total hip replacement or total knee replacement was present on the FLR, as this would have given erroneous measurements for coronal alignment. Subjects who were unable to progress as their joint space was already too narrow at baseline (< 1 mm) were excluded from the study, as their disease was too severe to be considered early to moderate knee OA.

Information regarding the acquisition of LLRs within OAI is publicly available [15]. Weight-bearing FLRs were taken of both lower limbs simultaneously with toes perpendicular to the film, the femoral epicondyles were kept parallel to the cassette and knees were kept fully extended whilst distributing the participants’ weight evenly.

A flow chart outlining the selection of the cohort is given in Fig. 1 and the baseline demographic details of the cohort are outlined in Table 1.

**Table 1:** Characteristics of the study cohort

Characteristic	Study cohort (*n* = 955)
Age (mean; SD)	60.7 (± 8.9)
Sex (% female)	57%
BMI (kg/m^2^; mean)	29.9
Race (% Caucasian)	69.8%
Co-morbidities (% none)	70.5%
Smoker (%)	7.2%
KL grade (%)	1 = 17% 2 = 50% 3 = 33%

### Outcomes

Symptom progression was defined as an increase in WOMAC score (symptom worsening) of > 9 points at 24 months compared to baseline [1]. Subjects with severe symptoms at baseline (WOMAC > 91 points) who could not progress by more than 9 points were defined as progressing if their symptoms were sustained (WOMAC > 91 points) at 24 months. WOMAC scores were normalized to have a 0–100 range.

Structural progression was defined as joint space narrowing (JSN) progression in the medial compartment (> 0.7 mm) [8].

### Radiographic assessment

**Fig. 2:**
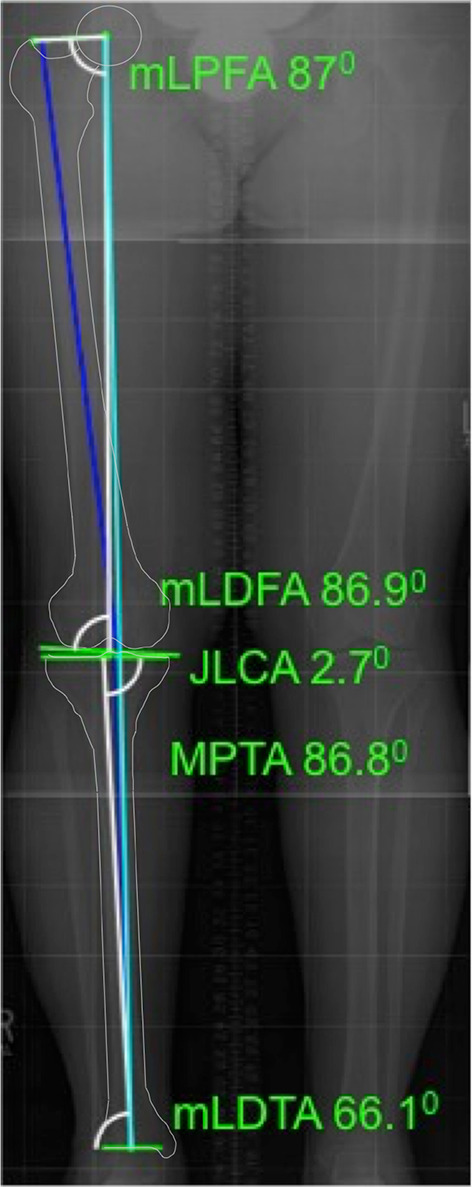
Alignment variables were obtained using Medicaid^®^ Classic (Hectec GMBH) for all individuals within the cohort. Mechanical alignment measurements included weight-bearing axis (WBA), mechanical lateral proximal femoral angle (mLPFA), mechanical lateral distal femoral angle (mLDFA), medial proximal tibial angle (MPTA), mechanical lateral distal tibial angle (mLDTA) and joint line convergence angle (JLCA). The light blue line indicates the weight-bearing axis expressed as a percentage of the medial–lateral tibial plateau (35.3% in this plan). NB: the outline of the femur and tibia has been highlighted in white to aid interpretation of this figure

All available FLRs were viewed as Digital Imaging and Communications in Medicine (DICOM) files and analysed using the Osteotomy Module within Medicaid^®^ Classic (Hectec GMBH) a surgical planning tool with high interrater reliability [17]. It generates mechanical alignment measurements including weight-bearing axis (WBA), mechanical lateral proximal femoral angle (mLPFA), mechanical lateral distal femoral angle (mLDFA), medial proximal tibial angle (MPTA), mechanical lateral distal tibial angle (mLDTA) and medial joint line convergence angle (JLCA_med) (Fig. 2).

### Observer reliability

A single observer (JP) reviewed all the FLRs in the cohort. Intraclass correlation coefficients (ICCs) were performed on readings of the first 20 limbs performed by the same observer (JP) after a 6-week interval (intra-observer reliability) and by another orthopaedic registrar trained in the use of Medicaid^®^ Classic (Hectec GMBH) (inter-observer reliability). ICCs were then repeated for readings of these 20 limbs made after the first 100, 200 and 500 LLR readings to identify any observer drift (JP).

Excellent intra-observer reliability was demonstrated with intraclass correlation coefficients > 0.90 for all alignment variables measured at baseline compared to those measured after the first 100 readings. There was no evidence of any observer drift with ICCs being maintained at > 0.80 after the first 200 and first 500 readings were performed. Excellent inter-observer reliability was also demonstrated (> 0.80) across all readings.

### Statistical analysis

#### Alignment variables by gender and baseline KL grade

Differences in alignment variables (mLPFA, mLDFA, MPTA, mLDTA) by gender and baseline KL grade were determined using generalised estimating equations (GEEs).

#### Alignment variables that predict overall alignment (e.g. valgus or varus)

The hip–knee–ankle angle (HKA) was converted to a binary outcome to separate those with valgus alignment (HKA > 0°) and those with varus alignment (HKA < 0°) [18]. Logistic regression models with GEEs were used to account for the potential correlation of observations that may arise from knees belonging to the same individual. The GEEs were used to identify which of the alignment variables (mLPFA, mLDFA, MPTA, mLDTA) predicted whether a subject was in varus or valgus alignment. Standardised coefficients were calculated to enable an assessment of how many standard deviations the HKA changed, per standard deviation increase in the alignment variable. In doing so, it was possible to determine which of the individual alignment variables, if any, had the greatest effect on the HKA.

#### Alignment variables and their effect on progression

Logistic regression models, with GEEs, were used to identify the baseline variables that predict symptom or structural progression at 24 months. Variables of interest were selected a priori: age, gender, BMI, number of co-morbidities, smoking status, race, employment status, and previous history of knee surgery. In addition, the individual mechanical alignment measurements were included in the statistical model: WBA, mLPFA, mLDFA, MPTA, mLDTA (Fig. 2).

Adjustments were made to some variables to facilitate their incorporation into the statistical models and subsequent interpretation. Employment status was converted from a categorical variable with four possible outcomes into a binary outcome (paid work vs not in paid work). Smoking status was converted from a categorical variable with four possible outcomes to a binary outcome (any smoking history vs no smoking history). The majority of participants in the OAI cohort reported their ethnic origin as black or Caucasian (97.1%). For this reason, the variable for race was simplified to a binary outcome (Caucasian vs non-Caucasian).

Each variable was added to the model in turn to assess its direct effect on the outcome of interest. Multivariate analysis, with all variables included, was then performed to explore and quantify the influence of these variables on OA progression. All models were adjusted for baseline JSN and baseline WOMAC score. Relationships between predictor variables and outcome were expressed as odds ratios (OR) and a *p* value < 0.05 was considered statistically significant. The independent variables included in the model were tested for collinearity using the variance inflation factor (VIF).

#### Multinomial regression analysis

Outcome groups were determined based on symptomatic worsening (WOMAC > 9 points) [1] and structural progression (JSN > 0.7 mm) [8]. Multinomial regression was then performed to determine the effect of predictor variables on the likelihood of an individual progressing to one of three outcome groups compared to the likelihood of progressing to a fourth referent group:

1. Non-progressor—no symptom worsening and no structural progression at 24 months (referent group).
2. Structural progressor—no symptom worsening but structural progression at 24 months.
3. Symptom progressor—symptom worsening but no structural progression at 24 months.
4. Structure and symptom progressor—symptom worsening and structural progression at 24 months.

Results were expressed as relative risk ratio (RRR) and *p* values of < 0.05 were considered statistically significant.

All statistical analyses were performed using Stata 13.1 (StataCorp. 2013. Stata Statistical Software: Release 13. College Station, TX: StataCorp LP).

## Results

955 individuals with 1,327 knees were included in the study.

**Fig. 3:**
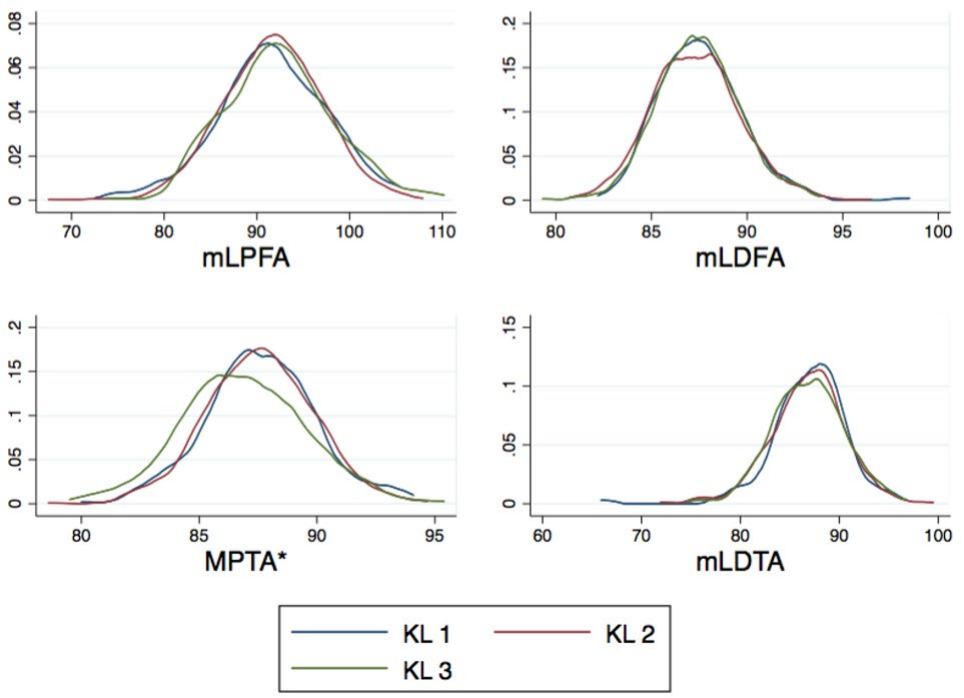
Kernel density plot graphs illustrating the differences in alignment variables observed by baseline KL grade. *p ≤ 0.05

Differences in alignment variables by gender and baseline KL grade are outlined in Table 2 and Fig. 3, respectively.

**Table 2:** The difference in alignment variables by gender is summarised

	Males (*n* = 535)		Females (*n* = 792)	
	Mean	SD	Mean	SD
mLPFA	91.8°	5.4	92.0°	5.8
mLDFA*	87.5°	2.0	87.3°	2.4
MPTA*	86.6°	2.3	87.9°	2.4
mLDTA*	86.2°	4.0	87.3°	3.5
JLCA_med	1.3°	1.6	1.1°	1.9

*Indicates a significant difference by gender (*p* < 0.05)

Standardised coefficients revealed that the scale of effect on varus and valgus alignment was similar for both MPTA and mLDFA (see Table 3).

**Table 3:** The influence of alignment variables on overall alignment is summarised

Alignment	Predictor	Odds ratio	95% CI	*p* value	Standardised coefficient
Varus	mLPFA	1.03	0.99–1.08	0.15	0.02
	mLDFA*	3.63	2.57–5.12	< 0.001	0.58
	MPTA*	0.23	0.16–0.34	< 0.001	− 0.60
	mLDTA*	1.11	1.04–1.19	0.003	0.03
Valgus	mLPFA	0.97	0.93–1.02	0.20	− 0.005
	mLDFA*	0.27	0.19–0.39	< 0.001	− 0.58
	MPTA*	4.44	2.91–6.77	< 0.001	0.61
	mLDTA*	0.90	0.84–0.97	0.005	− 0.03

*p ≤ 0.05

No alignment variables predicted symptom worsening at 24 months. Predictors of symptom worsening (WOMAC > 9 points) at 24 months included WOMAC score (*p* < 0.001) and BMI (*p* = 0.014) at recruitment. For every one unit increase in baseline BMI, the odds of developing worsening knee pain at 24 months increased by 6%. For every one unit increase in baseline WOMAC, the odds of developing worsening knee pain at 24 months decreased by 3% (see Table 4).

**Table 4:** Multivariate regression analysis to determine the influence of all baseline variables on symptom worsening (WOMAC > 9 points) and structural progression (JSN > 0.7 mm) over 24 months

Variable of interest	Symptom worsening (WOMAC > 9 points)			Structural progression (JSN > 0.7 mm)		
	Odds ratio	95% CI	*p* value	Odds ratio	95% CI	*p* value
Baseline WOMAC*	0.97	0.95–0.98	< 0.001	1.01	1.00–1.02	0.16
Baseline JSN	0.95	0.80–1.13	0.53	1.06	0.89–1.27	0.50
Age	0.99	0.96–1.02	0.41	1.01	0.98–1.04	0.56
Gender	1.46	0.90–2.37	0.13	1.18	0.70–1.98	0.53
BMI*	1.06	1.01–1.11	0.01	1.02	0.97–1.07	0.39
Co-morbidities	1.10	0.90–1.40	0.45	0.89	0.66–1.16	0.36
Race	0.76	0.47–1.24	0.27	1.15	0.67–1.96	0.62
Employment	0.63	0.38–1.05	0.07	1.22	0.70–2.12	0.49
Smoker	1.25	0.82–1.92	0.30	1.30	0.82–2.06	0.27
Previous knee surgery	1.19	0.70–2.00	0.52	0.97	0.56–1.69	0.91
mLPFA	1.03	0.99–1.06	0.18	1.02	0.98–1.06	0.39
mLDFA	0.92	0.84–1.01	0.09	1.05	0.95–1.16	0.35
MPTA**	1.05	0.96–1.15	0.31	0.79	0.71–0.88	< 0.001
mLDTA	1.02	0.96–1.08	0.59	1.02	0.96–1.08	0.50

*Indicates significant predictor of symptom worsening at 24 months (*p* < 0.05)

**Indicates significant predictor of structural progression at 24 months (*p* < 0.05)

The only variable that predicted structural progression at 24 months (JSN > 0.7 mm) was MPTA. The odds of JSN progression occurring at 24 months increased by 21% for every one degree decrease (more varus) in MPTA *(p* < 0.001) (see Table 4).

### Multinomial regression analysis

The cohort was split based on the four possible outcome groups. The majority of individuals were in the no-progression group (69.1%). This group was the referent group from which the RRR of a subject falling into one of the remaining three groups was calculated.

Only baseline WOMAC had a significant effect on the risk of being in the symptom worsening group (*n* = 158) relative to the referent group. For every one unit increase in the baseline WOMAC, the relative risk of being in the symptom worsening group decreased by a factor of 0.98 (*p* = 0.001).

Baseline WOMAC and MPTA had significant effects on the risk of being in the structural progression group (*n* = 195) relative to the referent group. For every one degree decrease in the MPTA (more varus), the relative risk of being in the structural progression group increased by a factor of 1.16 (*p* < 0.001). For every unit increase in the baseline WOMAC, the relative risk of being in the structural progression group increased by a factor of 1.01 (*p* = 0.02).

Baseline WOMAC, MPTA and gender had significant effects on the risk of being in the symptom and structural progression group (*n* = 57) relative to the referent group. For every one degree decrease in the MPTA (more varus), the relative risk of being in the symptom and structural progression group increased by a factor of 1.16 (*p* = 0.03). For every unit increase in the baseline WOMAC, the relative risk of being in the symptom and structural progression group decreased by a factor of 0.97 (*p* = 0.005). For female participants, the relative risk of being in the symptom and structural progression group, relative to the referent group, is expected to increase by a factor of 2.7 (*p* = 0.01).

### Collinearity

None of the variables in the multivariate model had a VIF greater than two and the mean of the VIF scores was close to one representing inconsequential collinearity for the model [16].

### Further analysis

Higher baseline KL grade was associated with a more varus MPTA. Baseline KL grade was not included in the statistical model, as it has a significant correlation with baseline JSN (Pearson correlation = − 0.4; *p* ≤ 0.001). To ensure that the predictive effects of MPTA are not the result of an unseen interaction between MPTA and baseline KL grade, the models were repeated with baseline KL grade exchanged for baseline JSN in the multivariate models for progression. The predictors of structural and symptom progression seen in the above results sections remained stable following this adjustment.

## Discussion

The most important finding of this study was that a varus MPTA was associated with significant structural progression in subjects with medial compartment knee OA. The odds of structural progression occurring at 24 months increased by 21% for every one degree decrease (more varus) in MPTA (*p* < 0.001). Whilst other factors contribute to both valgus and varus alignment (mLDFA, MPTA and mLDTA), they do not have a significant effect on structural progression. Subsequent, multinomial regression identified that for every one degree decrease in MPTA, the relative risk of being in the symptom and structural progression group and the structural progression group, relative to the referent group, increased by a factor of 1.16.

Several studies have reported on varus malalignment and its potent effects on structural progression in knee OA [6, 18]. Our study demonstrates that traditional alignment descriptions of varus, valgus and neutral are too simplistic and a more detailed assessment of coronal alignment is required. Hirschmann et al. have described functional knee phenotypes that acknowledge individual variations in alignment of the proximal tibia and distal femur and recommend that such phenotypes be used to facilitate an individualised approach to TKA surgery [12]. Our study highlights the importance of these individual alignment variables and their influence on knee OA progression.

This study challenges the observations made by other groups. Cooke et al. compared the alignment of 127 patients with symptomatic varus OA to 75 varus aligned healthy individuals [7]. Significant differences were observed in distal femoral geometry between the two groups leading the authors to conclude that it was distal femoral alignment that drove the development of medial compartment OA. This cross-sectional study did not have follow-up data from which to confirm this finding and it is not supported by our study.

No association was made between MPTA and symptom worsening at 24 months. A follow-up of 24 months may have been insufficient to detect any effect of individual alignment variables on symptom worsening at 24 months. A high baseline WOMAC score was, however, associated with reduced odds of symptom worsening at 24 months. Subjects for our cohort were selected based on their experiences of pain over the preceding 12 months, as such baseline WOMAC scores are likely to reflect an extreme measurement in terms of knee pain and subsequent regression to the mean [4] as time progresses.

Our study has other noteworthy limitations. The exclusion of certain variables could be a potential source of bias. Subjects with a FFD of more than 10 degrees were excluded from the study. A high FFD may in itself be associated with progression and by excluding this variable we may have biased the sample towards a risk group that is not related to FFD. The exclusion of those subjects who had evidence of bony attrition (*n* = 4) or arthroplasty surgery (*n* = 27) on their FLR may have selected out some important individuals with rapidly progressive OA. This reflected the fact that the OAI cohort did not have FLRs performed until at least 12 months after recruitment. As such, a participant may have progressed to end-stage disease before any assessment of coronal alignment was possible.

The participants in this study were selected to reflect patients who find themselves in the treatment gap. The average age of the cohort was 60.7 years and the mean MPTA was 87.4° (± 2.4), similar to that reported in a healthy population described by Bellemans et al. [87.04° (± 2.07)] [2]. Younger age patients (< 45 years) are not represented in this study and whilst some severe varus deformity will be present, the cohort as a whole shows very little variation in alignment. By contrast, a prospective study of individuals undergoing high tibial osteotomy with an average age of 47.5 years had a mean proximal tibial angle of 81.44° (± 4.51) [3]. Rates of progression in these younger patients with more severe proximal tibia vara would be of interest and has not been fully delineated.

Our cohort included subjects with symptomatic mild to moderate knee OA (KL grade 1, 2 and 3). This heterogeneous group was chosen as they reflect patients with symptomatic knee osteoarthritis and structural changes related to osteoarthritis that are not generally considered suitable for knee replacement surgery. Identifying risk factors for progression in this group are of interest as they have implications for surgical intervention.

Despite these limitations, this study has many strengths. The study population is large (*n* = 955; kn = 1327) and the statistical analysis was established a priori.

Proximal tibial alignment has clinical relevance. In the context of varus deformity, the proximal tibia can be corrected with a surgical intervention such as an osteotomy. Bonnin and Chambat reported superior clinical outcomes for patients undergoing osteotomy for medial OA for patients with a constitutional varus deformity of the proximal tibia [5]. This study supports the use of a high tibial osteotomy in the presence of proximal tibia vara, as it is this deformity that is driving structural progression over 24 months.

## Conclusion

This study has confirmed that whilst malalignment is a significant predictor of structural progression in symptomatic early knee OA, it is specifically the alignment of the proximal tibia that is associated with this phenomenon. Traditional alignment descriptions using the hip–knee–angle oversimplify coronal alignment. Future studies that aim to observe the effect of an intervention on structural progression of knee osteoarthritis will need to consider the effect of these individual alignment variables.
